# High prevalence of *pfmdr1* and *pfpm2* amplification and *pfmdr1* haplotypes in *Plasmodium falciparum* isolates circulating in Ghana

**DOI:** 10.1186/s12936-026-06075-9

**Published:** 2026-08-01

**Authors:** Abdul Rashid Kassim, Priscilla Akuba Younge, Ignatus Nunana Dorvi, Samirah Saiid, Mona-Liza Ebiere Sakyi, Osumanu Ahmed, Albert Yao Kudakpo, Stephen Tetteh Addo, Silas Nkansah Yeboah, Charles Mensah, Isaiah Debrah, Collins Misita Morang’a, Dominic S. Y. Amuzu, Gordon A. Awandare, Yaw Aniweh, Lucas Naam-Kayagre Amenga-Etego

**Affiliations:** 1 West African Centre for Cell Biology of Infectious Pathogens (WACCBIP), University of Ghana, Accra, Ghana; 2 Department of Biochemistry, Cell and Molecular Biology, College of Basic and Applied Sciences, University of Ghana, Accra, Ghana; 3 Department of Biology, University of Virginia, Charlottesville, VA USA

**Keywords:** Copy number polymorphism, *Plasmodium falciparum*, Plasmepsin, Multidrug resistance, Haplotypes

## Abstract

**Background:**

In Ghana, artemether-lumefantrine (AL), and artesunate-amodiaquine (AS-AQ) are the main first-line ACTs for uncomplicated malaria, with dihydroartemisinin-piperaquine (DHA-PPQ) as an alternative treatment regimen. Copy Number Polymorphisms (CNPs) in *Plasmodium falciparum* multidrug resistance 1 (*pfmdr1*) and plasmepsin 2 (*pfpm2*) genes, as well as specific *pfmdr1* haplotypes have been implicated in resistance to AL and DHA-PPQ partner drugs.

**Methods:**

This study characterised *pfmdr1* and *pfpm2* CNPs and *pfmdr1* haplotypes in 900 clinical malaria samples across three transmission zones in Ghana. Using a SYBR Green-based Quantitative real-time Polymerase Chain Reaction (qPCR) assay, followed by targeted amplicon sequencing.

**Results:**

Successful *pfmdr1* and *pfpm2* amplifications were detected in 32.0% (n = 288) and 27.8% (n = 250) isolates respectively. No significant difference in the prevalence of these gene amplifications was observed across the three eco-epidemiological zones (*pfmdr1*: p = 0.888; *pfpm2*: p = 0.081). Furthermore, a weak positive correlation was observed between *pfmdr1* and *pfpm2* copy numbers (ρ = 0.196), suggesting limited co-selection of these loci. *Pfmdr1* haplotype analysis at codons N86Y, Y184F, and D1246Y revealed a high prevalence (55.6%) of the NFD haplotype linked to lumefantrine selection. Interestingly, this haplotype allele was equally prevalent among isolates carrying single or multiple gene copy numbers.

**Conclusion:**

Taken together, this study observed increased prevalence of *pfmdr1* and *pfpm2* gene amplification in Ghana. Also, a high frequency of the single mutant NFD *pfmdr1* haplotype suggesting ongoing selection pressure potentially driven by widespread AL use in Ghana. In addition, *pfpm2* CNP has been associated with piperaquine resistance. These findings provide a critical baseline for ongoing molecular surveillance as an early warning for ACT partner drug resistance in Ghana.

**Supplementary Information:**

The online version contains supplementary material available at https://doi.org/10.1186/s12936-026-06075-9.

## Background

Ghana is a high-burden malaria country in Africa, where malaria transmission is perennial, with intensity varying across three distinct eco-epidemiological zones [1]. In 2024, Ghana reported approximately 6.7 million cases, contributing 2.4% of the global malaria burden and an estimated 11,635 deaths [2], highlighting the persistent public health impact of malaria in the country. Key national malaria control interventions include the use of long-lasting insecticide-treated bed nets (LLINs), indoor residual spraying (IRS), and seasonal malaria chemoprevention (SMC) [3].

Treatment for malaria in Ghana historically in the 1950s transitioned from traditional herbal preparations such as *Cryptolepis sanguinolenta* (Ghanaian quinine) and *Azadirachta indica* (neem) to the widespread use of chloroquine (CQ), following their demonstrated cost-effectiveness [4–6]. However, CQ resistance emerged in Southeast Asia and subsequently spread globally, prompting policy changes in endemic countries, including Ghana [1, 7, 8 ]. In 2005, Ghana switched to artemisinin-based combination therapies (ACTs) for the treatment of uncomplicated malaria, adopting multiple first-line treatments initially with artemether-lumefantrine (AL) and artesunate-amodiaquine (AS-AQ) and later dihydroartemisinin-piperaquine (DHAP) as an additional alternative [9].

While ACTs have been instrumental in reducing malaria morbidity and mortality, their efficacy is now threatened by the emergence and spread of resistant parasites. Artemisinin partial resistance, associated with mutations in the propeller domain of the *PfKelch13* gene, is well-established in Southeast Asia and has been confirmed in several East African countries, including Rwanda, Uganda, Ethiopia, Eritrea, and Tanzania [10–15]. Studies indicate that the *P. falciparum* plasmepsin 2 (*pfpm2*) and multidrug resistance 1 (*pfmdr1*) genes play key roles in mediating antimalarial drug resistance [16, 17 ]. Variations in copy number and mutations within these genes have been associated with reduced susceptibility to piperaquine, lumefantrine, and mefloquine [18–20].

The *pfpm2* gene product is the enzyme plasmepsin 2, an aspartic protease that plays a central role in the degradation of host haemoglobin within the parasite’s digestive vacuole [21]. Piperaquine, an ACT partner drug, is thought to disrupt heme detoxification and hemozoin formation in the digestive vacuole [22, 23 ]. However, its exact mechanism of action remains unclear [24]. Overexpression of *pfpm2* due to gene amplification has been strongly associated with piperaquine resistance [25]. However, piperaquine resistance is complex. Increased *pfpm2* copy number alone does not appear to confer resistance. Instead, it is opined that a multigenic background, combining *pfpm2* and *pfpm3* amplification, together with mutations in *pfcrt* such as T93S, F145I, I218F, H97Y and C350R contributes to piperaquine resistance [26–30].

The *pfmdr1* gene encodes *Pgh1* (P-glycoprotein homolog 1), an ATP-binding cassette (ABC) transporter located on the digestive vacuole membrane of the parasite. This transporter controls the uptake and efflux of a range of antimalarial drugs, including mefloquine, lumefantrine, amodiaquine, and artemisinin derivatives, within the parasite by transporting solutes from the parasite cytosol into the digestive vacuole [31, 32 ]. *Pfmdr1* amplification increases drug sequestration in the digestive vacuole, thereby decreasing drug accumulation and efficacy within the parasite [33].

The evolution of *pfmdr1* and *pfpm2* CNPs carries significant implications for drug efficacy in malaria endemic settings such as Ghana. Although these markers have been studied extensively in Southeast Asia, however, nationwide data on prevalence, spatial distribution, and co-occurrence of *pfmdr1* and *pfpm2* copy number polymorphisms in Ghana remains limited. Furthermore, the *pfmdr1* haplotype (N86Y-Y184F-D1246Y) variant have been associated with selection by artemether-lumefantrine and decrease parasite susceptibility to lumefantrine. This study aims to determine the prevalence and distribution of *pfmdr1* and *pfpm2* CNPs across Ghana’s eco-epidemiological zones, characterise associated *pfmdr1* SNPs and haplotypes, and investigate correlations between these genetic markers. By establishing baseline prevalence for these molecular markers in contemporary parasite populations, this work sought to generate data to guide national malaria control decisions that would preserve the longevity of ACTs in Ghana.

## Methods

### Study site and sample collection

**Fig. 1:**
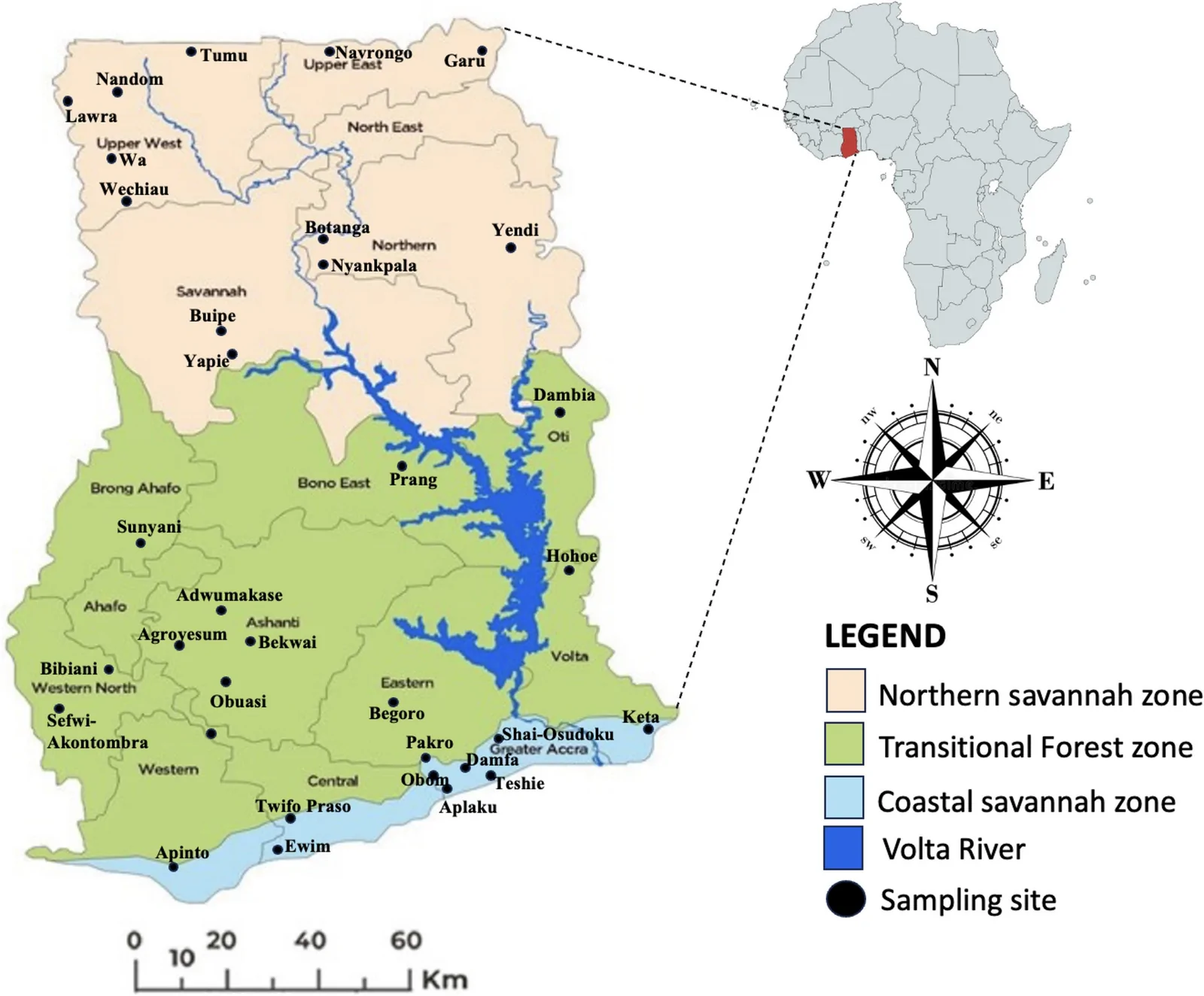
Map of Ghana showing the various eco-epidemiological zones and sentinel sites included in the study. Ghana is situated in West Africa at approximately 7.95˚ N and 1.02˚ W. The country shares borders with Togo to the east, Burkina Faso to the north, the Ivory Coast to the west, and the Gulf of Guinea to the south. The different zones: Northern Savannah (light brown), Transitional Forest (green), and Coastal savannah (sea blue) with the stretch of the Volta River (blue). Regional boundaries have been indicated by thin lines with the sample sites shown with the black bullet-point with the names of the communities

A retrospective cross-sectional study was conducted, utilising archived dried blood spot (DBS) samples collected from 2019–2023 as part of an ongoing nationwide malaria molecular surveillance program in collaboration with the Ghana National Malaria Elimination Program (NMEP) of Ghana and the West African Centre for Cell Biology of Infectious Pathogens (WACCBIP), University of Ghana. Samples were collected from sentinel health facilities strategically located across Ghana’s three malaria eco-epidemiological zones, namely Coastal Savannah, Transitional forest, and Northern Savannah (Fig. 1). These zones differ in climatic conditions, vector ecology, and malaria transmission intensity [34]. The Northern Savannah zone is characterised by high transmission with marked seasonality, peaking during the rainy season from May to October. The Forest zone experiences moderate to high transmission year-round, with peaks typically occurring from April to July and September to November. On the other hand, the Coastal Savannah zone has relatively low to moderate transmission intensity and is considered the zone with the lowest endemicity among the three [34]. Samples were collected from patients presenting with uncomplicated malaria, screened with malaria RDT, and confirmed by microscopy.

### Sample processing, DNA extraction and quantification

Venous blood (approximately 300 µL) was spotted onto Whatman No. 3 filter paper to create six dried blood spots (DBS) per sample (patient), which were air-dried, individually packaged in sealed ziplock bags with desiccant, and stored at 4 °C until processing. Genomic DNA was extracted from a single 6 mm DBS punch per sample using the QIAamp DNA Investigator Kit (QIAGEN, Germany) according to the manufacturer’s protocol with a few changes in buffer volumes to increase DNA yield. DNA was eluted in 50 µL of AE buffer and stored at -80 °C. DNA was quantified using Invitrogen™ Qubit™ Flex Fluorometer with the Qubit™ 1X dsDNA High Sensitivity (HS) Assay Kit (Thermo Fisher Scientific, USA).

### Pfmdr1 and Pfpm2 gene copy number analysis

Copy number polymorphisms for *pfmdr1* and *pfpm2* were quantified using SYBR Green-based quantitative real-time Polymerase Chain Reaction (qPCR) assay adapted from established protocols with minor optimizations [35, 36 ]. Each 10 µL reaction contained 5 µL of 2 × Universal qPCR Master Mix (New England Biolabs, USA), 0.25 µL each of 10 µM forward and reverse primers (*pfmdr1*; forward- 5′-TGT GTT TGG TGT AAT ATT AAA GAA CA-3′, reverse- 5′-ACA TAA AGT CAA ACG TGC ATT T-3′ or *pfpm2*; forward- 5′-TGG TGA TGC AGA AGT TGG AG-3′, reverse- 5′-TGG GAC CCA TAA ATT AGC AGA-3′), 2.5 µL nuclease-free water, and 2 µL of 2 ng/µL template DNA. Reactions were performed in a singleplex duplicate format, using an Applied Biosystems™ QuantStudio™ 5 Dx Real-Time PCR System (Applied Biosystems, Thermo Fisher Scientific, USA). The thermal conditions consisted of an initial hold at 50 °C for 2 min, denaturation at 95 °C for 15 min, followed by 40 cycles of 95 °C for 15 s and 60 °C for 30 s. A melt curve analysis step (95 °C for 15 s, 60 °C for 1 min, 95 °C for 1 s) was included to verify amplicon specificity. *P. falciparum* reference strain 3D7 was used as the calibrator for the relative quantification (2^-∆∆Ct) method, where ∆∆Ct = (Ct_target_—Ct_β-tubulin_)_sample_ − (Ct_target_ − Ct_β-tubulin_)_3D7_ and was included in every run. The Dd2 strain, known to have multiple copies of *pfmdr1* was used as a positive control for *pfmdr1*. The *P. falciparum* β-tubulin gene (forward-5′-TGA TGT GCG CAA GTG ATC C-3′, reverse- 5′-TCC TTT GTG GAC ATT CTT CCT C-3′) was used as the endogenous control. Samples with copy number estimate ≥ 1.5 were classified as having gene amplification (multiple copies), while those below this threshold were considered single copy. Inter-run variation was monitored using 3D7 ΔCt values as plates were repeated if 3D7 ΔCt deviated by > 0.3 cycles from baseline. The coefficient of variation (CV) for 3D7 ΔCt across all accepted runs was below 5%. Assays were repeated if the Ct value exceeded 35 or the standard deviation between duplicates was > 0.25.

### *Pfmdr1* SNP genotyping and haplotype determination

Following copy number estimations, amplicon sequencing was performed on the same *P. falciparum* clinical isolates to identify SNPs in the *pfmdr1* gene at codons 86, 184, and 1246. Parasite DNA was first enriched using selective whole-genome amplification (sWGA) as described by Oyola et al. [37]. The enriched DNA was further amplified in a two-step PCR targeting a custom panel for drug resistance loci, including *pfmdr1*, and barcodes for unique identification. The resulting libraries were pooled, size-selected using AMPure XP beads (Beckman Coulter), and quantified via qPCR with the KAPA Library Quantification Kit (Roche). Sequencing was performed on an Illumina MiSeq platform using MiSeq Reagent Kit v2 300 cycles (Illumina, San Diego, CA). Raw reads were demultiplexed, trimmed for quality, and aligned to the *P. falciparum* 3D7 reference genome (PlasmoDB v68) using BWA-MEM. Variant calling at target codons was performed with Clair3 version 1.1.1, and samples with > 10 × coverage at each codon were retained for analysis. Haplotypes were constructed based on the combined allele calls at codons 86, 184, and 1246. Detailed information on the *P. falciparum* amplicon toolkit protocol can be found here [38].

### Data analysis

All statistical analyses and visualisations were performed using the open source R statistical software (version 4.3.1). Genotyping of field isolates was scored as wild type, mutant, or mixed (i.e., heterozygous infections harboring both ref/alt alleles) The Chi-square test was used to compare the prevalence of gene amplification (single vs. multiple copies) across eco-epidemiological zones for each gene. Spearman’s rank correlation coefficient was used to assess associations between *pfmdr1* and *pfpm2* copy numbers, parasite density, and patient age. Differences in haplotype frequencies between eco-epidemiological zones and between single and multiple-copy isolates were evaluated using Fisher’s exact test or Chi-square test as appropriate. Statistical significance was defined as p < 0.05.

## Results

### Clinical and demographic characteristics

Of 900 samples screened, copy number analysis was successfully performed for *pfmdr1* in 899 (99.8%) samples, with a geometric mean parasite density (GMPD) of 34,349.6 [30,978.9–38,087.0]. For *pfpm2*, copy number analysis was successfully estimated for 898 (99.8%) samples, with a GMPD of 34,287.8 [30,921.5–38,020.5]. Patient ages ranged from 3 to 89 years, with a mean age of 13.9 years. A total of 897 samples yielded CNPs in both genes, with co-amplification observed in 10.9% (98/897).

### Prevalence of *pfmdr1 *and *pfpm2* copy number polymorphism

**Fig. 2:**
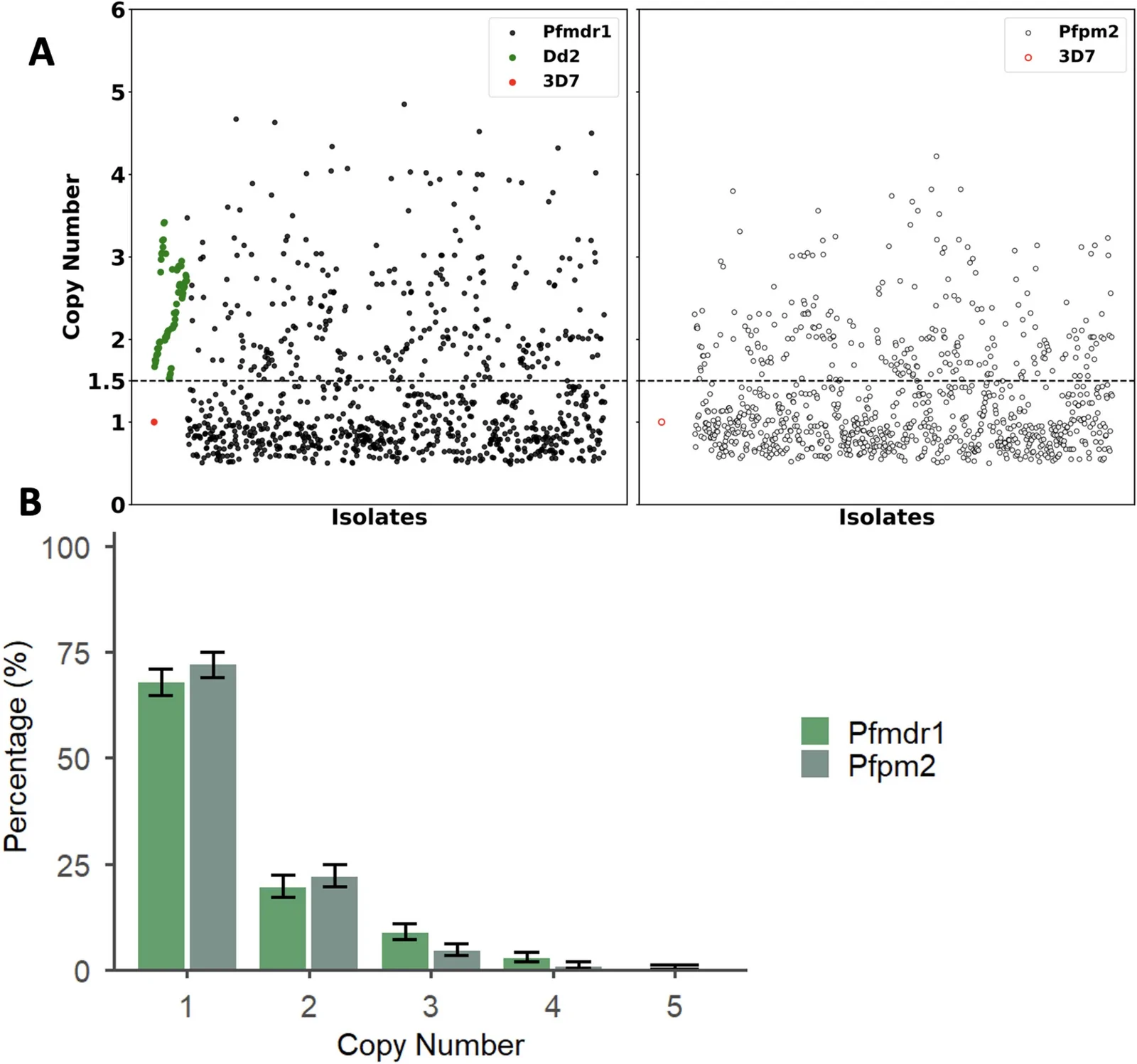
Scatter plot showing the distribution of *pfmdr1* and *pfpm2* gene copy number in clinical isolates from Ghana. **A** The first box shows the copy number of *pfmdr1* for each of the clinical isolates (black dots) relative to 3D7 (red dot) and Dd2 (green dots) strains. The second box represents the copy number for *pfpm2* (grey dots) for each of the isolates, and 3D7 (red). Isolates with a copy number ≥ 1.5 (dotted lines, black (*pfmdr1)* and grey (*pfpm2)* are classified as having gene amplification, whereas those below this threshold have a single copy. **B** The percentage (%) distribution of the different copy number estimates (1,2,3,4,5) for *pfmdr1* (green) and *pfpm2* (light green). The red dots represent the 3D7 reference strain with a copy number of 1 for both *pfmdr1* and *pfpm2*, while the green dots represent Dd2 with a mean copy number of 2.35 (95% CI 2.22–2.48) for the *pfmdr1*. Panel B: The error bars represent a 95% confidence interval

In each qPCR run, the laboratory strains 3D7 and Dd2 were included as controls. The 3D7 calibrator was assigned a copy number of 1 for both genes, whereas Dd2 showed mean copy number of 2.35 [95% CI 2.22–2.48] for *pfmdr1*. Samples with copy number values below 1.5 were classified as single copy, whereas those ≥ 1.5 were classified as multiple copy (Fig. 2).

Of the 898 samples analysed for *pfmdr1*, 68.0% (611/899) were single copy, while 32.0% (288/899) showed gene amplification. Copy number estimates for this gene ranged from 0.5 to 4.9, with a mean of 1.4. For *pfpm2*, 72.2% (648/898) of isolates had a single copy, whereas 27.8% (250/898) showed gene amplification (Fig. 2). Copy number estimates ranged from 0.5 to 4.2, with a mean of 1.3. There was a significant difference in the distribution of copy number estimates between *pfmdr1* and *pfpm2* (p < 0.001), and also between the two genes at copy number 3 (p < 0.001) and copy number 4 (p = 0.004) (Fig. 2).

### Prevalence of *pfmdr1 *and *pfpm2* copy number polymorphism by eco-epidemiological zones

**Fig. 3:**
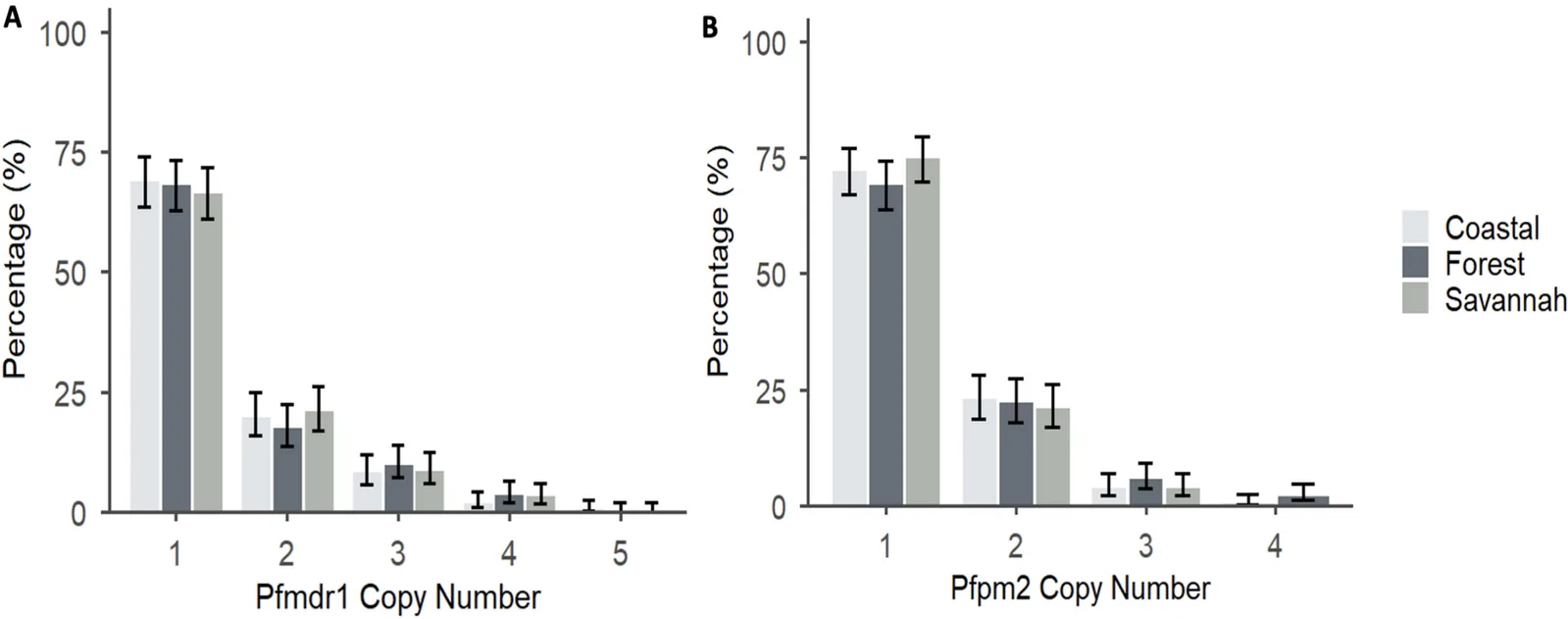
Distribution of *pfmdr1* and *pfpm2* copy number estimates across the three eo-epidemiological zones in Ghana. **A** The percentage (%) of the copy numbers of *pfmdr1* for the different zones; coastal (grey), forest (dark), savannah (light brown). **B** the percentage (%) distribution of the copy numbers of *pfpm2* across the different eco-ecological zones; coastal (grey), forest (dark), savannah (light brown). The error bars represent confidence intervals at 95%

By eco-epidemiological zone, the prevalence of *pfmdr1* gene copy number amplification was 31.0% (93/300) in the low-transmission coastal savannah zone, 31.7% (95/300) in the high-transmission forest zone, and 33.4% (100/299) in the high, but seasonal transmission northern savannah zone. In the coastal zone, *pfmdr1* copy number estimates ranged from 0.5 to 4.7, with a mean of 1.4. In the forest zone, *pfmdr1* copy number values ranged from 0.5 to 4.8, with a mean copy number of 1.4. The northern savannah zone recorded copy numbers between 0.5 and 4.5, with a mean of 1.4, and there was no significant difference in the distribution of copy number among the zones (p = 0.888) (Fig. 3).

For *pfpm2*, the prevalence of amplification was 27.8% (83/299) in the coastal savannah zone, 30.7% (92/300) in the forest zone, and 25.1% (75/299) in the northern savannah zone respectively. Copy numbers in the coastal zone ranged from 0.5 to 3.8, with a mean of 1.3, while in the forest zone they ranged from 0.5 to 4.2, with a mean of 1.3. The northern savannah zone recorded copy number estimates between 0.5 and 3.2, with a mean of 1.2, and there was no statistically significant difference in the distribution of copy number estimates between eco-epidemiological zones (p = 0.081) (Fig. 3).

### Correlation between *pfmdr1/pfpm2* CNPs, age, and parasitemia

Associations between *pfmdr1* and *pfpm2* gene copy numbers, parasite density, and age groups were assessed using Spearman’s rank correlation coefficient due to non-normal distribution of the copy number estimates. A weak positive correlation was observed between *pfpm2* and *pfmdr1* copy numbers (ρ = 0.196). Parasite density showed negligible correlation with gene copy numbers (*pfmdr1*: ρ = − 0.015; *pfpm2*: ρ = 0.008) and a strong negative correlation with age group (ρ = − 0.286). Age group showed a weak negative correlation with *pfmdr1* copy number (ρ = − 0.019) and *pfpm2* copy number (ρ = − 0.032) (Supplementary Fig. 1).

### Prevalence of *pfmdr1* single nucleotide polymorphisms

Of 899 samples, 741 (82.4%) were successfully sequenced and genotyped for single-nucleotide polymorphisms (SNPs) in the *pfmdr1* locus. Table 1 shows the prevalence of SNPs at three key codons in the *pfmdr1* locus (N86Y, Y184F, D1246Y). *Pfmdr1* codon 86 was predominantly wild type 98.4% (729/741), with few pure mutants, 1.1% (8/741) and mixed genotypes 0.5% (4/741). For *pfmdr1* codon 184, 25.1% (186/741) of isolates carried the wild-type allele (Y184), 57.2% (424/741) carried the mutant 184F allele, and 17.7% (131/741) of isolates were mixed. Furthermore, codon 1246 was completely wild type in 741 isolates (100%).

**Table 1:** Prevalence of single nucleotide polymorphisms (SNPs) in the *pfmdr1* locus in clinical isolates from Ghana

Mutations	Wild type n (%)	Mutant n (%)	Mixed n (%)
N86Y	729 (98.38)	8 (1.08)	4 (0.54)
Y184F	186 (25.10)	424 (57.22)	131 (17.68)
D1246Y	741 (100)	0 (0)	0 (0)

### Prevalence of mutant *pfmdr1* haplotypes

**Fig. 4:**
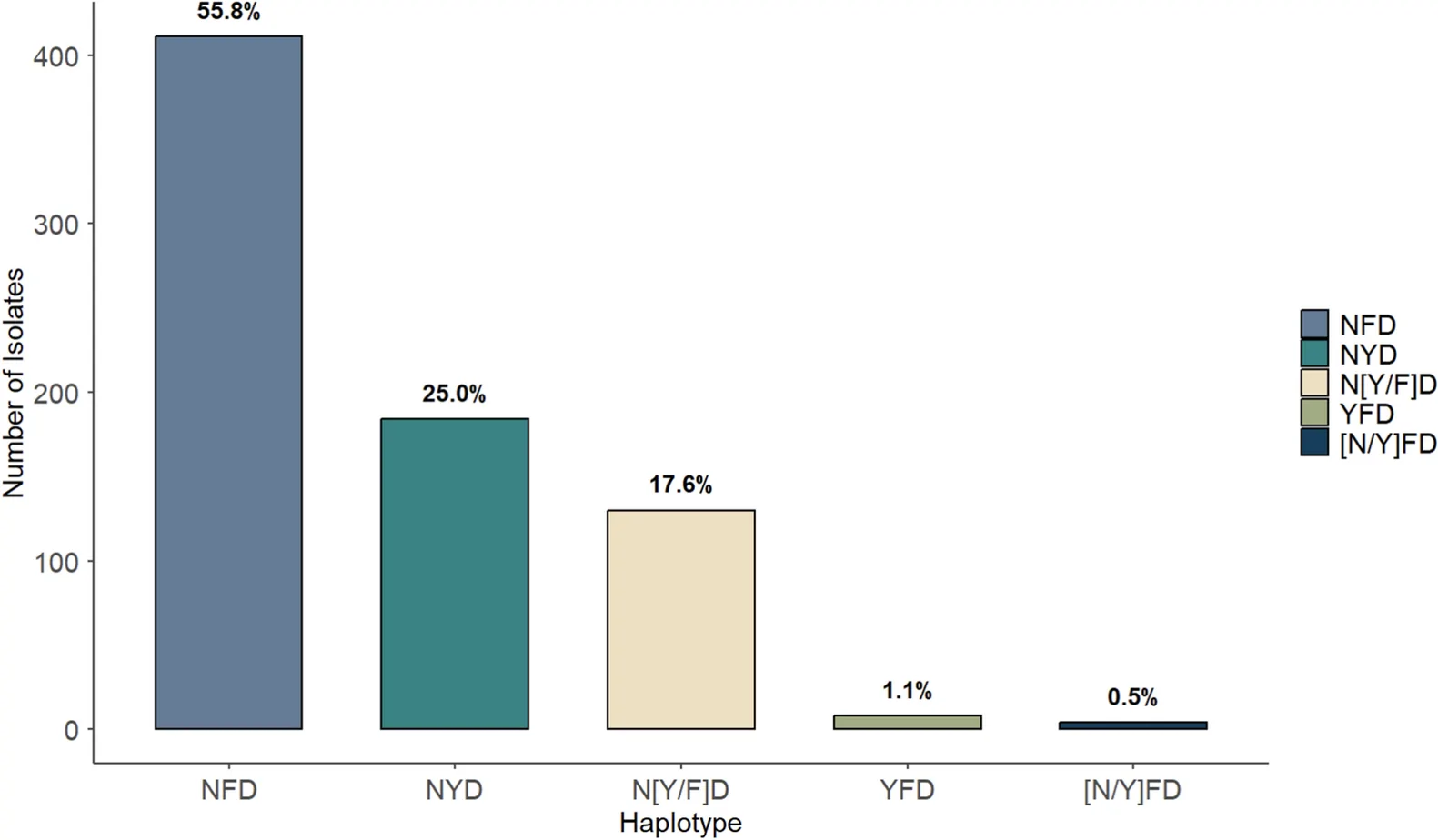
Distribution of *pfmdr1* haplotypes. The number of isolates as well as the percentage (%) (on top of each bar) bearing the different haplotypes with amino acid positions N86Y, Y184F, and D1246Y. The haplotypes studied includes NFD (dirty blue), NYD (green), N(Y/F)D (cream), YFD (green) and (N/Y)FD (blue-black)

*Pfmdr1* haplotype frequencies at codons 86, 184, and 1246 were successfully determined in 741 isolates. The single mutant NFD haplotype (N86/184F/D1246) was most common at a prevalence of 55.6% (412/741), followed by the wild-type NYD haplotype at 25.1% (186/741) and the double mutant YFD at 1.1% (8/741). Mixed haplotypes N[Y/F]D and [N/Y]FD were 17.7% (131/741) and 0.5% (4/741) respectively (Fig. 4).

### Prevalence of *pfmdr1* haplotypes by gene copy number

Haplotype frequencies were comparable between isolates with single and multiple *pfmdr1* copies. Among multiple-copy isolates (n = 377), the *pfmdr1* single mutant NFD haplotype was the most prevalent 57.3% (216/377), followed by the wild-type NYD haplotype 23.3% (88/377), mixed haplotype N[Y/F]D 18.0% (68/377), double mutant YFD haplotype 1.1% (4/377), and mixed haplotype [N/Y]FD 0.3% (1/377). Single copy isolates (n = 364) showed similar results, with the NFD being the most prevalent 53.8% (196/364), followed by NYD 26.9% (98/364), N[Y/F]D 17.3% (63/364), YFD 1.1% (4/364), and [N/Y]FD 0.8% (3/364). The differences in haplotype frequency between single and multiple-copy isolates were not statistically significant (p = 0.6498) (Supplementary Fig. 2).

### Prevalence of *pfmdr1* haplotypes by eco-epidemiological zones

**Fig. 5:**
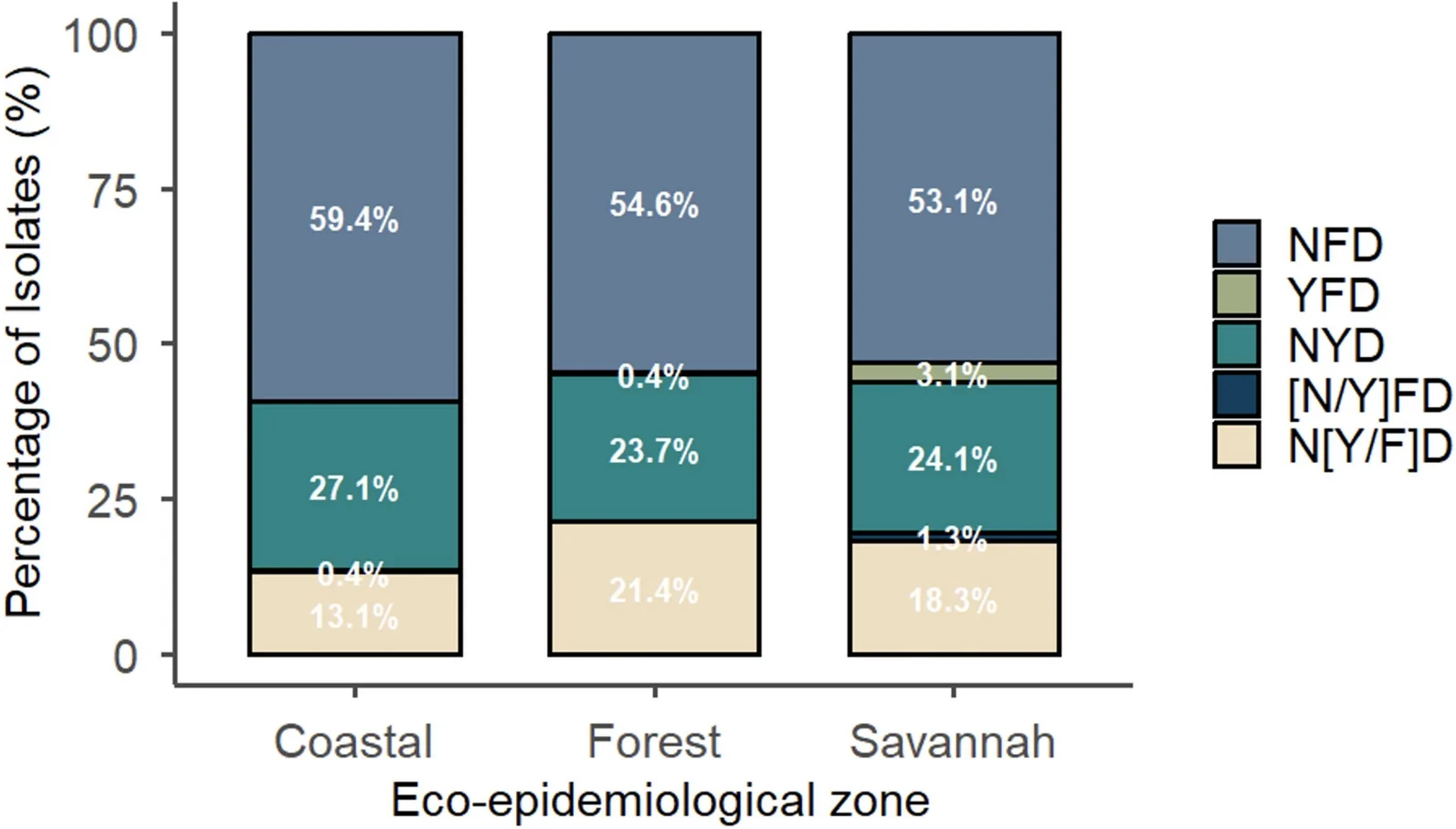
Distribution of *pfmdr1* haplotypes by eco-epidemiological zones. The percentage (%) representation of the different haplotypes segregated by the eco-ecological zones across Ghana; coastal, forest and savannah

The *pfmdr1* single mutant NFD haplotype was the most common across all eco-epidemiological zones, at a prevalence of 59.4% (149/251) in the coastal zone, 54.4% (143/263) in the forest zone, and 52.9% (120/227) in the northern savannah zone. The wild-type NYD haplotype was the second most common, ranging from 24.0% (63/263) in the forest zone to 27.1% (68/251) in the coastal zone. The mixed N[Y/F]D haplotype was most frequent in the forest zone, at 21.3% (56/263), compared to the coastal zone at 13.1% (33/251) and the northern savannah zone at 13.7% (31/227) zones. The double mutant YFD haplotype occurred at lower prevalence in the forest zone at 0.4% (1/263) and the northern savannah zone at 3.1% (7/227) zones and was absent in the coastal zone. The prevalence of the mixed [N/Y]FD haplotype in the northern savannah zone at 1.3% (3/227) and the coastal zone at 0.4% (1/251). There was a statistically significant difference in haplotype frequencies between eco-epidemiological zones (p = 0.0048) (Fig. 5).

## Discussion

The emergence and spread of resistance to artemisinin-based combination therapies (ACTs) represent a major threat to malaria control in endemic regions. Partner drugs are essential for the success of ACTs by sustaining parasite clearance, enhancing treatment durability, mitigating resistance development, and reducing malaria transmission [39, 40 ]. Monitoring the efficacy of partner drugs is critical for sustaining global malaria control efforts, as these would determine Africa’s transition from artemisinin partial resistance to full blown resistance to ACTs [41]. This study provides contemporary molecular surveillance data on CNPs in *pfmdr1* and *pfpm2*, along with *pfmdr1* single-nucleotide polymorphisms (SNPs) and haplotypes genotyped in *P. falciparum* isolates collected from clinical sources across Ghana’s three eco-epidemiological zones.

We observed high rates of amplification of *pfmdr1* and *pfpm2*, highlighting the circulation of parasites with amplified expression at these loci. However, such gene amplification events remain substantially lower than levels reported in Southeast Asia [32, 42 ]. Interestingly, we observed a higher *pfpm2* amplification compared to the 20.8% previously reported in Ghana [17]. Within the broader African continent, our study reports a higher prevalence of *pfpm2* amplification than in Mali (11%), Tanzania (10%), Gabon (11.3%), and Mozambique (12.5%), and approaches the levels observed in Burkina Faso (30.5%), our northern neighbor and Uganda (33.9%) [43–45]. This geographic variation likely mirrors differences in local drug pressure, particularly the deployment and duration of piperaquine-based therapies. The notable rise in Ghana could signal increasing selection for *pfpm2* amplification, potentially due to the growing use of DHA-PPQ or cross-resistance linked to other partner drugs.

The relatively low *pfpm2* amplification observed here aligns with the limited historical use of piperaquine-containing drugs in Ghana and supports the continued efficacy of DHA-PPQ, which is currently being deployed as an alternative ACT [35]. While increased copy number of plasmepsin genes has been linked to reduced piperaquine susceptibility and dihydroartemisinin-piperaquine treatment failure in Southeast Asia [46–48], current evidence suggests that *pfpm2* amplification alone does not fully account for the resistance phenotype [26, 27, 49 ]. Piperaquine response is influenced by a broader parasite genetic background, including *pfpm3* amplification, mutations in other resistance-associated loci such as *pfcrt*, and variations in local drug pressure [23, 28 ]. This broader perspective is particularly relevant for African parasite populations, as recent data from Mali demonstrated that decreased post-treatment protection following repeated dihydroartemisinin-piperaquine use was associated with shorter time to reinfection and a higher frequency of infections with parasites that amplified *pfpm3* [50]. Consequently, the 27.8% *pfpm2* amplification observed in Ghana should be viewed as an early molecular signal that warrants continued monitoring, rather than as evidence of established piperaquine resistance.

In Ghana, the prevalence of *pfmdr1* amplification was reported as 4% in 2003–2004, rising to 27% in 2005–2006, then declining to 9% in 2007–2008, and subsequently increasing again to 18% in 2010 [16]. Our current findings of 32.0% *pfmdr1* amplification indicate a continued upward trend in recent years, potentially reflecting sustained selective pressure from widespread AL use in Ghana. Across the African continent, the prevalence of *pfmdr1* amplification exhibits considerable heterogeneity, often correlating with local drug-use patterns [51–56].

CNPs in *pfmdr1* and *pfpm2* were observed at comparable frequencies across Ghana’s three eco-epidemiological zones despite marked differences in malaria transmission intensity and parasite prevalence between these zones. This spatial homogeneity might be caused by high gene flow between zones, driven by human migration, local travel, and vector dispersal, which effectively erodes genetic isolation. In addition, the nationwide deployment of AL and DHA-PPQ as part of the primary multiple first-line treatment approach likely exerts similar selective pressure on parasite populations across all eco-epidemiological zones, further contributing to the lack of significant differences across these transmission landscapes.

The weak positive correlation between *pfmdr1* and *pfpm2* copy numbers (ρ = 0.196) indicates limited co-variation of these loci within the sampled parasite population. This finding aligns with the distinct selective pressures exerted by different ACT partner drugs. Specifically, *pfmdr1* amplification is primarily linked to lumefantrine and mefloquine selection, while *pfpm2* amplification is driven by piperaquine pressure. Clinical and genomic studies have demonstrated that *pfpm2* and/or *pfpm3* amplification frequently occurs in parasites carrying *pfmdr1* single copy background, highlighting the independent evolutionary trajectories of these markers [57, 58 ]. Moreover, piperaquine resistance is increasingly understood as a multigenic trait involving complex interactions between *pfpm2*, *pfpm3*, and mutations in *pfcrt* among others. *Pfcrt* mutations such as T93S, H97Y, F145I, I218F, and C350R have been shown to confer piperaquine resistance even in the absence of *pfpm2* amplification, and epistatic interactions between these loci are believed to be critical for high-level resistance [20, 23, 59,27 ]. The lack of association between CNPs and parasite density or age further implies that these genetic changes do not confer a clear fitness advantage in terms of growth or transmissibility under natural infection conditions.

Furthermore, *pfmdr1* codon 86 was highly conserved, with a higher number (> 95.0%) of isolates retaining the wild-type allele (N86). This contrasts with patterns reported in East Africa and parts of Asia, where the mutant 86Y allele is often prevalent and linked to amodiaquine and legacy chloroquine resistance [32, 60, 61 ]. The near absence of 86Y mutants in Ghana likely reflects the country’s historical transition to multiple ACTs from chloroquine and the subsequent expansion of chloroquine sensitive parasites [16]. Also, the 184F mutation, which has been linked to reduced susceptibility to lumefantrine and other ACT partner drugs, was highly prevalent, occurring in 57.2% of isolates [62]. The 184F mutation forms part of a complex genetic background, that may correlate with other loci to mediate reduced susceptibility to several ACT partner drugs. Our findings highlight the selective pressure exerted by AL on the *pfmdr1* gene in parasite isolates circulating across all transmission transects in Ghana.

At the haplotype level, the single mutant NFD variant was dominant across all zones and equally prevalent in single and multi-copy isolates, reinforcing its role as a widespread adaptive genotype in *P. falciparum* strains in Ghana. This is in line with previous reports from Ghana and other African countries where the *pfmdr1* NFD haplotype is highly prevalent [44, 63, 67 –]. Thus far, the body of knowledge suggests this haplotype confers a fitness advantage under AL exposure. Notably, the lack of association between the NFD haplotype and increased *pfmdr1* copy number aligns with observations from Cambodia, where the NFD haplotype was not directly linked to increased *pfmdr1* copy number [68].

These findings though robust, some limitations in the current study need be acknowledged. The relatively small sample size may not allow extrapolation or the interpretation of the data across the entire parasite population in Ghana due to possible sampling biases related to transmission seasonality, patient demographics, or clustering of treatment choices within eco-epidemiological zones. Also, the absence of direct phenotypic drug-susceptibility data (e.g., ex vivo assays or clinical efficacy outcomes) limits the ability to link observed CNPs and haplotypes to drug resistance, as resistance marker prevalence may not necessarily correlate with phenotypic resistance [64]. Furthermore, our study focused on *pfpm2* amplification without concurrent assessment of *pfpm3* CNPs or *pfcrt* resistance-associated mutations, which backgrounds may be useful for data interpretation. These findings should be regarded as a molecular surveillance indicator rather than direct evidence of piperaquine resistance. Future studies should therefore employ a multi-marker approach that includes *pfpm2*, *pfpm3*, and key *pfcrt* loci to provide a robust early warning for piperaquine resistance.

## Conclusion

The increased prevalence of *pfmdr1* and *pfpm2* amplifications, as well as the high frequency of the lumefantrine-selected *pfmdr1* NFD haplotype, suggests emerging molecular signatures associated with reduced susceptibility of *P. falciparum* isolates in Ghana to AL and DHA-PPQ following over two decades of exposure. Therefore, the integration of molecular surveillance into national therapeutic efficacy monitoring is essential in harnessing the value of these markers for malaria control decision making. Such integration would enable the early detection of declining drug susceptibility, guide timely policy adjustments, and ultimately preserve treatment efficacy to advance Ghana’s malaria control and elimination agenda.

## Supplementary Information

Supplementary material 1. Supplementary Figure 1: Correlation between *pfmdr1* and *pfpm2* gene copy number estimates, parasite density, and age using Spearman’s rank correlation coefficient. The magnitude and direction of the correlation are indicated by the colours and values on the y-axis, and the correlation coefficients are shown above the diagonal. Supplementary Figure 2: Distribution of pfmdr1 gene haplotypes among isolates with single and multiple gene copies. The percentage (%) representation of the different haplotypes segregated by single and multiple copy number isolates.

## Data Availability

The raw sequence and genotype data generated and the associated metadata for this study is available at the WACCBIP High-performance Computing cluster. The genotype datasets are available upon reasonable request to the corresponding author.
